# Antioxidant Activity and Healthy Benefits of Natural Pigments in Fruits: A Review

**DOI:** 10.3390/ijms22094945

**Published:** 2021-05-06

**Authors:** Wang Lu, Yuan Shi, Rui Wang, Deding Su, Mingfeng Tang, Yudong Liu, Zhengguo Li

**Affiliations:** 1 Key Laboratory of Plant Hormones and Development Regulation of Chongqing, School of Life Sciences, Chongqing University, Chongqing 401331, China; lwang4901@163.com (W.L.); 18306012148@163.com (Y.S.); wangrui076023@163.com (R.W.); dedingsu@163.com (D.S.); 2 Center of Plant Functional Genomics, Institute of Advanced Interdisciplinary Studies, Chongqing University, Chongqing 401331, China; 3 Institute of Agricultural Quality Standard and Testing Technology, Chongqing Academy of Agricultural Sciences, Chongqing 401329, China; 1987tfming@163.com

**Keywords:** carotenoids, flavonoids, anthocyanidins, fruits, antioxidant, health benefits

## Abstract

Natural pigments, including carotenoids, flavonoids and anthocyanidins, determine the attractive color of fruits. These natural pigments are essential secondary metabolites, which play multiple roles in the whole life cycle of plants and are characterized by powerful antioxidant activity. After decades of research and development, multiple benefits of these natural pigments to human health have been explored and recognized and have shown bright application prospects in food, medicine, cosmetics and other industries. In this paper, the research progress of natural fruit pigments in recent years was reviewed, including the structural characteristics and classification, distribution in fruits and analysis methods, biosynthetic process, antioxidant capacity and mechanism, bioaccessibility and bioavailability, and health benefits. Overall, this paper summarizes the recent advances in antioxidant activity and other biological functions of natural fruit pigments, which aims to provide guidance for future research.

## 1. Introduction

Eating fresh fruits is a major source for people to intake natural nutrients; beyond that, fruit can also be fermented or processed into fruit wine, fruit juice, preserved fruit and other products for consumption. Fruits contain a variety of natural pigments, which are secondary metabolites with important biological activities [1,2]. In addition, fruit color is also an important commodity quality that determines the consumers’ choice. These pigments play essential roles in plant growth and development, photosynthesis, attracting pollinators and seed carriers, and resisting biotic and/or abiotic stresses [3]. The main pigments in fruits include carotenoids contributing red, yellow, and orange (for example, apricot and tomato); flavonoids contributing yellow (for example, citrus); anthocyanidins contributing red, purple, and blue (for example, grape and blueberry). These pigments have powerful antioxidant activities and multiple health benefits, such as delaying aging, repairing the nervous system, anti-atherogenicity, anticancer, and anti-inflammation [4,5,6]. Studies on the natural fruit pigments and their antioxidant activities and functional mechanisms are of guiding significance for targeted breeding and help improve the nutritional and commodity quality of fruits. In this paper, based on the relevant studies in recent years, the structural characteristics and classification, distribution in fruits, biosynthesis process, antioxidant capacity and functional mechanism, bioaccessibility and bioavailability, and health benefits of fruit natural pigments are reviewed.

## 2. Carotenoids

Carotenoids, a class of irreplaceable micronutrients in the human diet, are widely found in various bacteria, fungi, algae, and plants. So far, more than 800 natural carotenoids have been found, with colors of red, orange, yellow, etc. [7]. In plants, carotenoids are a category of lipophilic natural pigment stored in chloroplast and chromoplast membranes. They make plant organs, including fruits, appear red, yellow and/or orange [8]. Carotenoids also exist in green tissues as photosynthetic pigments. They can capture light energy and deliver it to the chlorophyll a in the excited state, which performs the function of transforming light energy [9]. Moreover, carotenoids are effective active oxygen scavengers with provitamin activity and can reduce oxidative stress in the human body. They have an outstanding effect on chronic diseases, preventing cardiovascular and cerebrovascular diseases, eye diseases, osteoporosis and cancer [10]. There are abundant and diverse carotenoids in fruits, as more than 100 kinds have been identified [8].

### 2.1. Chemical Structure and Classification of Carotenoids

Carotenoids are a class of tetraterpenoid fat-soluble polyene pigments, and they are C40 terpenoid compounds, and the derivatives are composed of eight isoprenoid units. The polyene skeleton is the most prominent feature of carotenoids, and most of the molecules have bilaterally symmetric and varied numbers of conjugated double bonds, generally 3–15, which confer carotenoids a strong ability of light harvesting and oxidation-reduction [11]. The structural characteristics enable carotenoids to be precursors and converted into a variety of active substances in the living body through various pathways [8,11]. In plants, most carotenoids exist in the form of more stable trans isomers, which have strong lipophilicity and polarity, and can be either free in cells or esterified with fatty acids or complexed with sugars and proteins, which depend on the molecular structure [12]. Depending on the presence or absence of the oxygen atom, carotenoids can be roughly divided into xanthophylls (C_40_H_56_O_2_ or C_40_H_56_O) and carotenes (C_40_H_56_) [8]. Xanthophylls in plants mainly include lutein, zeaxanthin, capsanthin, β-cryptoxanthin, and so on. Carotenes in plants include α-carotene, β-carotene, γ-carotene, lycopene, phytoene, and phytofluene, and the first three are biosynthesis precursors of vitamin A [13]. Depending on whether there is a terminal ring, carotenoids can also be classified into cyclic and acyclic carotenoids [8].

### 2.2. Carotenoids in Fruits

Almost all fruits contain carotenoids. Some typical fruits that are rich in carotenoids are mentioned here, including goji berries (*Lycium barbarum*), tomato (*Solanum lycopersicum*), and apricot (*Prunus armeniaca*). Goji berries are recognized as a healthy food for both medicine and diet, the orange-red color of goji fruits is mainly determined by carotenoids. The dry wild goji fruits contain about 6 mg/g of carotenoids, of which zeaxanthin dipalmitate accounts for about 70–80%, which endowed the powerful ability to scavenge oxygen free radicals and active oxygen [14]. Tomatoes are important commercial crops, and the content of carotenoids is an important indicator of their quality. Tomato fruits contain at least twelve kinds of carotenoids, of which lycopene accounts for 70–90% of the total carotenoids, and β-carotene accounts for 5–26%. Lycopene confers tomato fruits with a strong antioxidant capability and high nutritional value [15]. Apricot fruits are also rich in carotenoids, and seven types of carotenoids have been identified in apricots. The content of *trans*-β-carotene is approximately 61.2 mg/kg (dry weight, DW), accounting for 39–65% of total carotenoids; 9-*cis*-β-carotene accounting for 15%; 3-*cis*-β-carotene, pre-xanthin, and β-cryptoxanthin accounting for about 10%; lutein accounting for 4%; zeaxanthin accounting for about 3%. To sum up, β-carotene makes up the predominant proportion of total carotenoids in apricot fruits, which makes it one of the best sources of β-carotene [16].

### 2.3. Biosynthesis of Carotenoids

Carotenoids are tetraterpenoids composed of eight isoprenes. There are two independent terpenoids biosynthesis pathways in plants, namely, the mevalonic acid (MVA) pathway in cytoplasmic and the 2-*C*-Methyl-d-erythritol-4-phosphate (MEP) pathway in plastid [8]. Both pathways can produce isopentenyl pyrophosphate (IPP) and dimethylpropenyl diphosphate (DMAPP), which are precursors of carotenoids biosynthesis [17,18]. Pyruvate and glyceraldehyde 3-phosphate (G3P) generate MEP under the catalysis of 1-deoxy-d-xylulose-5-phosphate (DXP) synthase (DXS) and DXP reductoisomerase (DXR) [19]. Additionally, DXS is the first rate-limiting enzyme, and MEP finally formed IPP and DMAPP after a series of enzymatic reactions [20].

Then, DMAPP and IPP (1:3) are condensed under the catalysis of geranylgeranyl pyrophosphate synthase (GGPPS) to produce a variety of direct precursors of carotenoids biosynthesis. Under the catalysis of phytoene synthase (PSY), two molecules of geranylgeranyl pyrophosphate (GGPP) undergo a condensation reaction to produce colorless linear phytoene [8]. PSY is the most critical rate-limiting enzyme in carotenoids biosynthesis; antisense inhibition of the tomato *SlPSY1* gene significantly reduced the carotenoid content in the fruit, resulting in yellow fruit at maturity [21]. Under the catalysis of phytoene dehydrogenase (PDS) and ζ-carotene dehydrogenase (ZDS), *cis*-lycopene is formed after two dehydrogenation reactions. Finally, under the catalysis of carotene isomerase (CRTISO), the conformation changes to produce pink-colored all-*trans* lycopene [3]. Another important point in the carotenoids’ metabolism process is the cyclization of lycopene. Lycopene cyclases (LCY-β and LCY-ε) catalyze the cyclization of both ends of lycopene to produce α-carotene and β-carotene, and α-carotene transforms into lutein under the catalytic action of β-carotene hydroxylase (BCH) and ε-cyclohydroxylase (ECH) [22]. Zeaxanthin goes through two consecutive reactions under the continuous catalysis of zeaxanthin cyclooxygenase (ZEP) and neoxanthin synthase (NSY) to synthesize violaxanthin and then further converts into neoxanthin. This is the final step of carotenoids biosynthesis [22].

Tomato is a model material for studying fruit ripening and metabolisms. In recent years, there have been many studies on transcriptional regulation of carotenoids’ biosynthesis and metabolism in tomatoes. RIN is one of the most deeply studied MADS-box transcription factors, and the tomato *rin* mutant cannot turn red normally and contains a small amount of lycopene or even no lycopene [23]. The expression of the *PSY1* gene is hardly detected in the *rin* mutant, and recent studies have shown that RIN directly binds to the *PSY1* promoter to regulate carotenoids biosynthesis in fruit [24]. In addition, several other members of the MADS-box transcription factor family also play important roles in the regulation of carotenoids biosynthesis, including SlTAGL1, SlMBP15, SlMADS1, and SlMBP8 [25,26,27,28]. Other transcription factors also participate in this process, but most of which indirectly regulate the carotenoids biosynthesis genes [29].

## 3. Flavonoids

Flavonoids belong to the polyphenol superfamily and are synthesized by plants and widely exist in stems, leaves, flowers, and fruits. Flavonoids are usually appeared yellow, light yellow, or white. Seed disseminators are attracted by the attractive color of fruits, which is prominently contributed by flavonoids. Plants produce flavonoids in response to various abiotic and biotic stresses, and flavonoids also participate in regulating plant growth and development [30]. There are a variety of flavonoids with rich contents in fruits, and more than 5000 different kinds have been identified from plants [31]. In recent years, more and more studies have reported multiple benefits of flavonoids on human health. Flavonoids have gradually become a research hotspot in biology, food science, medicine, and other fields.

### 3.1. Chemical Structure and Classification of Flavonoids

Flavonoids are a category of phenols with a basic C15 phenyl-benzopyrone skeleton formed by a C6-C3-C6 structure, and most contain the 2-phenylchroman core [32]. The typical flavonoid compound is a flavone, which was first identified from stems and leaves of the *Primula* plant [33]. The 2-phenylchroman core is constituted by two aromatic rings (A and B) linked by a central three-carbon chain that may or may not form a third ring (C). According to the connection position of B-ring (C-2, C-3 or C-4), most classes of this secondary metabolite known as flavonoids (flavones, flavonols, flavonones, and flavanols), isoflavonoids (isoflavones, isofavonones, isoflavonols, isoflavans, rotenoids, coumestans, pterocarpans, and isoflavenes) and neoflavonoids (arylcoumarins, neoflavenes, etc.), respectively [34]. Moreover, flavonoids can be divided into flavonols, flavones, flavanols (catechins), flavanones, anthocyanins, and isoflavonoids based on variations and saturation of the C-ring [35]. There are some special cases, including aurones, chalcones, and dihydrochalcones etc. are the primary product of flavonoids biosynthesis, which are also precursors of other classes [34]. For example, chalcone is not considered a true flavonoid due to the lack of the aromatic C-ring but is still considered a member of the flavonoid family [36]. On the other hand, various modifications of flavonoids are very common. Hydroxyl, methoxyl, methyl, isopentenyl, methylenedioxyl, benzyl, nitro, etc., are usually substituted on A-ring and/or B-ring. Glycosidated and methylated derivatives are the main forms of plant natural flavonoids. d-glucose, l-rhamnose, glucorhamnose, galactose, and arabinose can be formed glycosidic bonds with flavonoids at the C-3 or C-7 positions [37].

### 3.2. Flavonoids in Fruits

People in different regions have different dietary patterns, which was influenced by geographical factors [37]. However, no matter what kind of dietary habits, fruits play an indispensable role and are an excellent bank of dietary flavonoids. Flavonoids in berry fruits are mainly found in the peels and seeds, grape (*Vitis vinifera*), citrus fruit (family *Rutaceae*) and several tropical fruits mentioned here are typically fruits rich in flavonoids. Flavonoids have long been a mainstay of researches in grape and wine because this class of substances not only affects the color of the wine but also the aging capacity, bitterness, and astringency [38]. Anthocyanins, flavan-3-ols (tannins, generate proanthocyanidins), and flavonols are the main flavonoids found in grapes and their derivatives, and the first two kinds will be discussed in the next section. Flavonols only accumulate in the skin of grapefruit, quercetin, myricetin, and kaempferol occupy 90% of flavonols, while laricitin, isorhamnetin, and syringetin make up the remaining 10% [39]. Citrus fruit is an import bulk fruit all over the world, which is rich in a variety of physiologically active substances. Citrus polyphenols (including flavonoids) are mainly found in the peel, which accounts for 40–50% of the citrus fruit biomass [40]. Citrus flesh also contains many kinds of flavonoids, but the content is significantly lower than that in peels [41]. Citrus peel contains abundant glycosylated flavones and polymethoxylated flavones (PMFs) [42]. The glycosylated flavones in citrus include hesperidin, neoeriocitrin, poncirin, dydimin, narirutin, naringin, diosmin, and isorhoifolin [43,44]. The PMFs are a unique class of highly methoxylated flavonoids, which exist almost exclusively in citrus peels (Lu et al., 2020). Nobiletin and tangeretin are the most abundant PMFs in citrus, accounting for 25.1% and 16.9% of the total content, respectively [42,45]. As a by-product of the citrus industry, citrus peels have been widely used in the food, pharmaceutical, and cosmetic industries [41]. In addition, tropical fruits also contain abundant flavonoids. From an evaluation of fruit by-product water extract (FWE) by HPLC-DAD, passion fruit presented rutin (7.0 mg/L FWE) and quercitin (4.0 mg/L FWE). Acerola fruit only presented rutin (8.0 mg/L FWE). Additionally, mango fruit presented rutin (29.0 mg/L FWE), quercitin (4.0 mg/L FWE), and epicatechin (2.0 mg/L FWE) [46].

### 3.3. Biosynthesis of Flavonoids

Flavonoid biosynthesis has been widely studied and well understood. Flavonoids are produced in the cytoplasm, catalyzed by a series of enzymes that belong to shikimic acid and the phenylpropanoid pathway and the acetate pathway, and then are transported to vacuoles for storage [47,48]. Each step of flavonoids biosynthesis is catalyzed by a specific enzyme family that is conserved in higher plants [49]. Phenylalanine, at the beginning of flavonoid biosynthesis, is deaminated by phenylalanine ammonia lyase (PAL) and hydroxylated by cinnamate-4-hydroxylase (C4H). Then, it is converted to 4-coumarate [50]; 4-coumarate contains a carboxyl group, which could be activated by 4-coumarate-CoA ligase (4CL) to produce 4-coumarate-CoA. Then, 4-coumarate-CoA acts as a substrate for subsequent enzymes, which also makes it become a branching point for either phenolic acids or flavonoids. The synthesis of chalcone is considered to be the beginning of flavonoids’ biosynthesis. Under the action of chalcone synthase (CHS), three molecules of malonyl-CoA, the product of the acetate pathway, are condensed with one molecule of 4-coumarate-CoA to form A-ring and B-ring of typical flavonoids C6-C3-C6 skeleton. Additionally, the C-ring is formed by chalcone catalyzed by chalcone isomerase (CHI), and flavanone (naringenin) is formed in this step. A branch is arisen here for the production of flavone, and a double bond is formed between C-2 and C-3 of the naringin C-ring catalyzed by flavone synthase (FNS). Returning to the trunk, naringenin is hydroxylated by flavonone-3-hydroxylase (F3H), flavonoid-3′-hydroxylase (F3′H), or flavonoid-3′-5′-hydroxylase (F3′5′H) to form dihydrokaempferol, dihydroquercetin, or dihydromyricetin, respectively. Subsequently, they are reduced by dihydroflavonol reductase (DFR) to form leucocyanidin, leucopelargonidin and leucodelphinidin, respectively. These leucoanthocyanidins are then catalyzed by flavonol synthase (FLS) to form kaempferol, quercetin, or myricetin, respectively [47,48,49,50].

There have been many studies on the regulation of flavonoid biosynthesis. R2R3-MYB transcription factors occupy the core position in the regulatory network, which could directly regulate the key biosynthesis genes [51]. In the transgenic *Arabidopsis thaliana* that is heterologously expressed *VvMYBF1*, the accumulation of flavonoids was significantly increased [52]. Another study also showed that overexpression of *VvMYBF1* in the transgenic *Arabidopsis thaliana* induced the expression of *AtPAL*, *AtCHS*, *AtCHI*, *AtF3H*, *AtF3ʹH*, *AtFLS*, *AtDFR*, *AtLDOX,* and *AtUFGT* and enhanced the activity of corresponding enzymes [53]. In addition, basic helix-loop-helix (bHLH) and WD40 protein also participate in the regulation of flavonoids accumulation. The MYB-bHLH-WDR (MBW) protein complex efficiently regulates flavonoids accumulation [54]. Beyond that, other transcription factors are also involved in the regulatory network. For example, a recent study indicated that three citrus AP2/ERF subfamily members act as positive factors to promote the expression of *CitCHIL1*, which enhances the accumulation of flavanones and flavones [55].

## 4. Anthocyanidins

Given the prominent specificity and high nutritional value of anthocyanidins, they are discussed separately in this section. There are a large number of studies on anthocyanidins, which have different colors compared with other flavonoids. Anthocyanidins appear in different colors that are affected by the basic structure, co-pigmentation, metal ion complexation, and vacuole pH [4]. They make fruits and flowers appear in orange, red, purple, or blue and cooperate with other natural pigments to build gorgeous colors in plants. Proanthocyanidins (Pas) and anthocyanidins come from the same branch of flavonoids biosynthesis pathway, which mainly includes catechins, epicatechins, and their polymers, which are unique reddish-brown substances found in fruits [56]. They also determine the bitterness and astringency of the fruits and their secondary products [57]. So far, more than 600 different anthocyanidins have been identified and widely distributed in at least 27 families, 73 genera, and countless species [58].

### 4.1. Chemical Structure and Classification of Anthocyanidins

Anthocyanidins belong to a category of typical flavonoids, also contain the basic C6-C3-C6 skeleton. The unique color of anthocyanidins comes from their conjugated bond [4]. Cyanidin, pelargonidin, delphinidin, peonidin, malvidin, and petunidin are the most common anthocyanidins. Among them, cyanidin is the most abundant in fruits that accounting for about 50% [50]. Same as the flavonoids, the chemical diversity and bioavailability of anthocyanidins are contributed by the number and location of glucosides [59]. Anthocyanidins in fruit also exist mainly in the form of glycosylation [59]. In addition, acylated anthocyanins are also found in plants, which can be further divided into acrylated anthocyanin, coumaroylated anthocyanin, caffeoylated anthocyanin, and malonylated anthocyanin [59].

### 4.2. Anthocyanidins in Fruits

Anthocyanidins are widely distributed in a variety of fruits and are abundant in fruits, making them ready to obtain. Grapes are the most common fruit that rich in anthocyanins, and there are a large number of anthocyanins in grape skins and wine. Proanthocyanidins are mainly found in peels and seeds but very little in the pulp [60]. The quality of wine is mainly contributed by proanthocyanidins, which determine the astringency, browning, and turbidity [60]. Blueberry (*Vaccinium corymbosum*) is also the most representative and well-known fruit that rich in anthocyanins, about 22 kinds of anthocyanins have been detected in blueberry [61]. Wang et al. [62] measured and analyzed the metabolic profile of 14 varieties of blueberry; five anthocyanins including cyanidin (154.5–1001 µg/g FW, fresh weight), delphinidin (3.3–69.7 µg/g FW), malvidin (3.0–55.8 µg/g FW), petunidin (5.6–51.9 µg/g FW), and peonidin (4.0–164.3 µg/g FW) were identified by HPLC-PAD. Although the content of different kinds of anthocyanins in blueberry varies greatly, in general, blueberry contains very high levels of anthocyanins. Purple kiwifruit (*Actinidia species*) contains cyanidin and delphinidin [63]. Peng et al. used ultra-high performance liquid chromatography (UHPLC) and liquid chromatography/mass spectrometry analysis (LC-MS) to analyze anthocyanins in different varieties of purple kiwifruit at different development stages. According to their research, cyanidin-based anthocyanins and delphinidin-based anthocyanins were distributed in the pericarp and flesh, and the anthocyanins in the pericarp were higher than that in the flesh at different developmental stages [64].

### 4.3. Biosynthesis of Anthocyanidins

Leucoanthocyanidins (leucocyanidin, leucopelargonidin, and leucodelphinidin), which are derived from the flavonoid biosynthesis pathway, are common precursors of anthocyanidins and flavan-3-ols [48,49]. Anthocyanidin synthase (ANS), also known as leucoanthocyanidin dioxygenase (LDOX), catalyzes three leucoanthocyanidins into cyanidin, pelargonidin, and delphinidin, respectively. As substrates, leucoanthocyanidins and anthocyanidins are reduced to (+)-catechin and (−)-epicatechin under the catalytic action of leucoanthocyanidin reductase (LNR) and anthocyanidin reductase (ANR) [48,50]. Finally, anthocyanidins undergo structural modification, including glycosylation, acylation, and methylation under the catalytic action of UDP-glucose flavonoid-3-*O*-glucosyltransferase (UFGT), acyl transferase (AT), and methyl transferase (MT), respectively [49].

As a kind of important flavonoid, anthocyanidins biosynthesis is also tightly regulated by the evolutionarily conserved MBW complexes [54]. In tomato, SlAN2-like (SlMYB114) is a major transcriptional regulator that promotes anthocyanidins biosynthesis [65]. Our study also presented that overexpression of *SlMYB75* (*SlAN2*) in tomato results in high-level anthocyanidins in various tissues [66]. In apple, a long terminal repeat (LTR) retrotransposon was found to insert the upstream of the *MdMYB1* gene and enhance its expression and then activate anthocyanidins biosynthesis [67].

## 5. Antioxidant Capacity and Health Benefits

### 5.1. Antioxidant Mechanisms

Reactive oxygen species (ROS) are produced by oxidation reactions in organisms [68]. The biochemical imbalance caused by ROS may damage many biological macromolecules, including DNA, RNA, lipid, and protein, that lead to degenerative diseases such as multiple sclerosis, cancer and Alzheimer’s disease [69]. The characteristic structure of carotenoids is conjugated polyunsaturated double bond, and this particular chemical structure confers the ability to inhibit free radicals. The conjugated polyunsaturated double bond also confers carotenoids a lipophilic character and, thus, protects lipoproteins and cell membranes from free radical attack [70]. Flavonoids, including anthocyanidins, are capable of directly scavenging free radicals by donating the hydrogen atom or indirectly inhibiting free radicals by chelating free metal ions [71,72]. In addition, flavonoids are also closely related to the cellular oxidant-antioxidant enzyme system and inhibit some of the enzymes that produce ROS, including NADH oxidase, glutathione S-transferase, mitochondrial succinoxidase, and microsomal monooxygenase. On the other hand, flavonoids enhance the activity of antioxidant enzymes [71]. For example, hesperidin could enhance the activity of antioxidant enzymes, including superoxide dismutase (SOD), glutathione peroxidase (GPX), glutathione reductase (GRX), and catalase (CAT) [73], and quercetin could activate glyoxalase activity [74].

### 5.2. Antioxidant Capacity and Detection Methods

Different in vitro assay methods may generate different results when evaluating the antioxidant capacity of natural pigments in fruits. This is because a single analysis method cannot take into account the following factors: the diversity and complexity of the antioxidant reaction mechanism, the distribution effect of antioxidants in a heterogeneous system, and the influence of other components in the test system. Thus, multiple assays are often used simultaneously to measure antioxidant capacity objectively [75]. The following assays are commonly used to evaluate the antioxidant capacity of fruit pigments: 1,1-dipheny-1-2-picrylhydrazyl (DPPH), and 2,2-azinobis (3-ethylbenzothiazoline-6-sulphonic acid) (ABTS) free radical scavenging assay, total radical-trapping antioxidant parameter (TRAP), oxygen radical absorbance capacity (ORAC), and ferric reducing antioxidant power (FRAP). These reaction principles are essentially based on the ability of artificially produced free radical scavenging to evaluate the antioxidant capacity of the substance under test [76]. Besides, the total phenolic content (TPC) and total flavonoid content (TFC) also partly represent the antioxidant capacity [77].

The composition and content of natural pigments vary greatly among different fruits that caused significant differences in antioxidant capacity between different fruits, even in the same variety [46,62]. When people eat fruits or other related products, a variety of natural pigments with antioxidant properties intake, it seems more practical to study the overall antioxidant capacity. We summarize the detailed information of several common fruits on the main natural pigments and their overall antioxidant capacity in Table 1.

### 5.3. Health Benefits

The natural pigments in fruits are not only powerful antioxidants, but they also have a host of other health benefits. Many studies have reported the biological and pharmacological activities of carotenoids, flavonoids and anthocyanidins, including antioxidant, anticancer, antiviral, antibacterial, anti-inflammatory, antiallergic, antithrombotic, cardioprotective, hepatoprotective, neuroprotective, antimalarial, antileishmanial, antitrypanosomal, and antiamebial properties.

Carotenoids and their metabolites are supposed to have positive effects on several ROS-mediated diseases such as cardiovascular disease, osteoporosis, cancer, and myocardial infarction in smokers [105,106]. The epidemiological study indicated that carotene-rich foods and supplements could prevent prostate cancer to a certain extent [107]. However, there was also suggested that a high dose of β-carotene has no effect on preventing lung cancer in smokers [108]. The relationship between prostate cancer risk with carotenoids (such as α-carotene and β-carotene) has been studied for many years, but the results are mostly inconclusive [109,110]. In addition, carotenoids containing β rings with provitamin A activity can transform into retinol and play important roles in the visual system [109]. Other reports also showed that carotenoids could improve cognitive function [111] and act as skin photoprotectants with cosmetic benefits [8]. In a word, carotenoids are widely used in the food, pharmaceutical, and cosmetic industries due to the obvious health benefits.

Flavonoids have long been a research hotspot in phytology, food science, and nutriology due to their powerful antioxidant capacity and multiple health benefits. With the deepening of research, the health benefits of flavonoids have been gradually explored, including but not limited to antioxidant, anticancer, cardioprotective, neuroprotective, anti-atherosclerosis, antimutagenic, antiallergic, antitumor, and anti-inflammatory capabilities [42,45,68,101]. Although ROS is an important means of self-protection and immunity in cells, excessive ROS will damage biological macromolecules and eventually lead to cell death [112]. Antioxidants could scavenge free radicals that confer strong anticancer properties, and flavonoids could inhibit tumor cell growth [5]. Polymethoxylated flavones (PMFs) widely exist in citrus, which has been shown to be lethal to a variety of human cancer cell lines (DLA, MCF-7, A549, and HepG2) [45,113]. Multiple methoxy groups endow PMFs with the ability to penetrate cell membranes, allowing them to work directly within the target cancer cells [42]. Compared with vincristine (anticancer drug), the extract of citrus peel has less toxicity to normal cells, which means it can be added into food as nutraceuticals and cancer preventive agents [113].

Hyperlipidemia and hyperglycemia are common and frequently occurring diseases, which seriously threaten human health and are often difficult to be completely cured. They will cause a variety of complications, including cardiovascular and cerebrovascular disease, chronic kidney failure, and fatty liver disease [114,115]. Powerful health benefits of flavonoids make it an excellent supplement to the treatment of hyperglycemia, hyperlipidemia and their complications. Tangeretin and nobiletin can reduce serum cholesterol and triacylglycerol [116], possibly through inhibit sterol regulatory element-binding protein [45]. The mechanism underlying the antibacterial activity of antioxidants is still unclear. However, some studies have shown that there are three possible mechanisms for its antibacterial activity: outer membrane permeability, cytoplasmic leakage, and inhibition of nucleic acid formation [117,118]. Flavonoids can directly affect the growth of bacteria and inhibit their pathogenic activity [119], such as *Cutibacterium acnes* (lead to acne), *Streptococcus mutans,* and *Lactobacillus acidophilus* (lead to dental caries) [120].

On the other hand, independent studies have demonstrated the multiple human health benefits of anthocyanins in the diet [121,122]. These health benefits include inhibition of various cancers, protection of cardiovascular and nervous systems, and balance of metabolisms, such as blood glucose and lipids, which have been demonstrated in experimental models, such as in vitro, mammalian, and human clinical and epidemiological studies [123].

### 5.4. Bioaccessibility and Bioavailability

Fruit natural pigments exhibit strong antioxidant activity and multiple health benefits. However, whether these beneficial bioactive compounds can be effectively used by the human body depend on their bioaccessibility and bioavailability. Bioaccessibility indicates the proportion of ingested natural pigments that are absorbed by the intestine, and bioavailability indicates the number of natural pigments that enter the blood circulation of the human body after digestion and absorption. Only this part can finally play function or be stored in the human body [68]. The primary digestive mechanisms of natural pigments determine their bioaccessibility and bioavailability. In addition, the chemical properties also affect their bioaccessibility and bioavailability. However, the chemical properties of fruit natural pigments are more unstable than other nutrients, such as sugar and organic acids. Thus, as the stability is enhanced, so does the bioavailability [124].

Carotenoids are abundant in fruits, but their bioavailability is not as high as expected (10–65%) [125]. Carotenoids perform powerful physiological functions only after entering the human bloodstream. Before that, carotenoids are involved in a series of digestive processes and are eventually taken by intestinal mucosal cells and then enter the lymphatic system and circulation. Many factors work together in this process, including release from the food matrix, mass transfer phenomenon, and micelle formation [126].

The bioaccessibility of oral phenolic compounds (including flavonoids and anthocyanidins) is 5–10% [127]. Flavonoids in fruits usually exist as flavonoid glycosides and PMFs; the modification of these substituents has a great influence on the bioactivity of flavonoids and their bioavailability. The bioaccessibility and bioavailability of flavonoid glycosides depend on the aglycone, both in its type and position. The number and position of the methoxyl group of PMFs have a decisive effect on its biological activity [68]. With the increase of the methoxyl group, PMFs exhibit higher hydrophobicity and stronger biological activity. However, it is also a double-edged sword, a high-level hydrophobicity results in poor oral bioavailability, which limits its potential application [128]. In general, compared with flavonoid glycosides, PMFs have lower polarity and approximate planar structure, higher permeability to biofilm, and easier to enter into the blood circulation in vivo [42].

The bioaccessibility and bioavailability of anthocyanidins were generally considered to be low, even lower than other flavonoids commonly found in fruits [123]. The conclusion was drawn by previous studies only focused on the result that anthocyanidins content in the bloodstream was very low after intake [129,130]. More and more recent studies revealed the existence of anthocyanidins molecular intermediates, which retain the unique flavonoid C6-C3-C6 backbone structure and have the characteristics that include qualitative diversity, relatively high concentrations, and tenacity [123,131]. Therefore, the present evaluation method of anthocyanidins bioavailability may be not accurate, but more evidence is needed.

Flavonoids, especially anthocyanidins and proanthocyanidins, have much a lower antioxidant capacity than carotenoids [3]. However, due to the water-soluble characteristic, flavonoids are more easily absorbed by the human body than liposoluble carotenoids, which means higher bioaccessibility and bioavailability [80,130]. In addition, the contents of flavonoids in fruits were more abundant than that of carotenoids [3]. Therefore, the health function of flavonoids is more powerful. However, this does not mean that carotenoids are unimportant because some of their properties cannot be replaced, such as the prevention of certain cancers. Therefore, for the highest efficacy, reasonable and balanced intake of these pigment substances is necessary.

## 6. Conclusions and Perspective

Natural pigments of fruits are nontoxic, renewable, and easily available, which endow wide application prospects in the food, medicine, and cosmetics industries. The iterative progress of chromatography technology promotes the identification of natural pigments to be micro and refined, more and more compounds are discovered and understood. Now researchers have realized the importance of degradation and storage in the process of natural pigment accumulation, and this study was initiated with these two aspects. The antioxidant capacity and mechanism of natural pigments have been clarified to a certain extent, which provides a basis for better utilization of natural pigments.

There are still many gaps to be filled in the field of fruit natural pigments. For example, there are still many unclear nodes in the transcriptional regulatory network of natural pigments biosynthesis, and the study on post-transcriptional regulation is also rare. Moreover, the existing chemical methods are not accurate enough to determine antioxidant capacity, and technologies, such as simulation in vitro digestion, also need to be improved, which limits the study on antioxidant capacity and bioavailability. In addition, the compositions of natural pigments are complex, and some of them are degraded easily, which results in a low level of bioavailability. This brings great challenges to the effective use of natural pigments to serve human health. With the development of technology and further research, we hold the opinion that these problems will be solved gradually.

## Figures and Tables

**Table 1:** The antioxidant capacity and main antioxidant pigments of common fruits.

Fruit	Antioxidant Capacity	Methods	Main Pigments	References
**Tomato** (*Solanum* *lycopersicum*)	27.42 µmol TE/g DW	PCL	Carotenoids: lycopene, lutein, α-carotene, β-carotene Flavonoids: naringenin, kaempferol, quercetin	[78,79]
	32.37 µg DW	DPPH		
	13.32 ± 0.79%			
	10.51 ± 0.31 mg/100 g FW	TPC		
	8.02 ± 0.37 mmol/L 100/g FW	FRAP		
**Grape** (*Vitis* *vinifera*)	2605 ± 487 µmol TE/100 g FW	ORAC	Flavonoids: quercetin, kaempferol Anthocyanins: delphinidin, malvidin, cyanidin, petunidin, peonidin	[80,81,82,83,84,85]
	42.18 mmol TE/g (seed)			
	36.40 mmol TE/g (skin)			
	70.14 ± 1.54 mg of catechin/100 g	TPC		
	281.3 µmol TE/g (seed) 12.8 µmol TE/g (skin) 2.4 µmol TE/g (flesh)	TEAC		
	58.04 mol TE/100 g (seed)	FRAP		
	32 mmol Fe^2+^/L (juice)			
	16.8 to 92 mmol TE/g (seed)	DPPH		
	15.7 to 113.3 mmol TE/g (skin)			
	15 mmol TE/L (juice)			
**Blueberry** (*Vaccinium* *corymbosum*)	4521 µmol TE/100 g FW	ORAC	Flavonoids: quercetin glycosides, myricetin glycosides Anthocyanins: cyanidin glycosides, peonidin glycosides, delphinidin glycosides, malvidin glycosides, petunidin glycosides	[62,86,87,88]
	4669 µmol TE/100 g			
	1287 µmol Vit. C equiv./100 g FW	PSC		
	128 ± 30 µmol of QE/100 g (no PBS wash) 19.0 ± 4.7 µmol of QE/100 g (PBS wash)	CAA		
	5.88 ± 1.17 µmol/g FW	ABTS		
	7775.45 ± 1009.60 EC_50_—g FW/g	DPPH		
	99 ± 5.28 µg/mL (juice)			
	0.3336 ± 0.1219 µg/mL (juice)	MAO-A		
	0.1064 ± 0.03630 µg/mL (juice)	Tyrosinase		
	1.26 ± 0.109 µg/mg (juice)	Glucosidase		
	0.453 ± 0.07156 µg/mg (juice)	DPP-4		
**Blackberry** (*Rubus* *fruticosus*)	13.23 ± 1.37 µmol/g FW	ABTS	Flavonoids: kaempferol glycosides, quercetin glycosides, myricetin glycosides Anthocyanins: pelargonidin glycosides, cyanidin glycosides, peonidin glycosides	[86,88,89,90]
	2142.42 ± 125.64 EC_50_—g FW/g	DPPH		
	5905 µmol TE/100 g	ORAC		
	201.05 ± 4.69 µmol Fe^2+^/g DM (mature)	FRAP		
	241.06 ± 4.44 µmol Fe^2+^/g DM (fully mature)			
	20.35 ± 3.25 µM/g FW	TEAC		
**Strawberry** (*Fragaria* *ananassa*)	8348 ± 888 µmol TE/100 g FW	ORAC	Flavonoids: kaempferol glycosides, quercetin glycosides Anthocyanins: pelargonidin glycosides, cyanidin glycosides, peonidin glycosides	[81,86,88,91,92]
	4302 µmol TE/100 g			
	136 ± 18 µmol of QE/100 g (no PBS wash) 42.2 ± 3.3 µmol of QE/100 g (PBS wash)	CAA		
	7.87 ± 0.87 µmol/g FW	ABTS		
	3778.94 ± 333.88 EC_50_—g FW/g	DPPH		
	417.9 mg AA/100 g FW			
	2.15 mmol Fe^2+^/100 g FW	FRAP		
**Cherry** (*Prunus* *serotina*)	5945 ± 978 µmol TE/100 g FW	ORAC	Flavonoids: quercetin glycosides, myricetin glycosides, kaempferol Anthocyanins: cyanidin glycosides, peonidin glycosides, pelargonidin glycosides	[81,86,93,94]
	27.4 ± 4.1 µmol of QE/100 g (no PBS wash) 6.8 ± 0.8 µmol of QE/100 g (PBS wash)	CAA		
	8.83 ± 1.32 µmol/g FW	ABTS		
	50.03 ± 1.32 mg ascorbic acid eq. 100/g FW			
	6065.68 ± 563.46 EC_50_—g FW/g	DPPH		
**Peach** (*Prunus* *persica*)	2235 ± 278 µmol TE/100 g FW	ORAC	Carotenoids: β-cryptoxanthin, β-carotene Flavonoids: quercetin glycosides, isorhamnetin glycosides, kaempferol glycosides Anthocyanins: cyanidin glycosides	[81,95,96]
	1586 ± 51 µmol TE/100 g FW (nectarine)	ORAC		
	7.1 to 88.7 mg/serving (white-flesh nectarine)	DPPH		
	9.1 to 40.0 mg/serving (yellow-flesh nectarine)			
	19.6 to 107.3 mg/serving (white-flesh peach)			
	13.0 to 50.5 mg/serving (yellow-flesh peach)			
	14.4 to 105.5 mg/serving (white-flesh nectarine)	FRAP		
	15.9 to 49.5 mg/serving (yellow-flesh nectarine)			
	27.9 to 119.6 mg/serving (white-flesh peach)			
	19.0 to 72.2 mg/serving (yellow-flesh peach)			
**Plum** (*Prunus* *salicina*)	5661 ± 440 µmol TE/100 g FW	ORAC	Carotenoids: β-cryptoxanthin, β-carotene Flavonoids: quercetin, myricetin, eriodictyol Anthocyanins: cyanidin glycosides	[81,96,97,98]
	1181 to 2366 µmol TE/100 g (summer varieties)			
	1510 to 3444 µmol TE/100 g (autumn varieties)			
	33.5 ± 8.6 µmol of QE/100 g (no PBS wash) 12.9 ± 0.1 µmol of QE/100 g (PBS wash)	CAA		
	27.4 to 61.1 mg/serving	DPPH		
	203 to 730 µmol TE/100 g (summer varieties)			
	132 to 554 µmol TE/100 g (autumn varieties)			
	40.5 to 127.2 mg/serving	FRAP		
	863 to 4215 mmol AAE/100 g (summer varieties)	Haemolysis		
	2012 to 12449 mmol AAE/100 g (autumn varieties)			
**Apricot** (*Prunus* *armeniaca*)	21.68–69.78% inhibition	DPPH	Carotenoids: β-cryptoxanthin, lutein, β-carotene, γ-carotene. zeaxanthin Flavonoids: rutin, quercetin 3-rutinoside, kaempferol 3-rhamnosyl-hexoside and quercetin 3-acetyl-hexoside Anthocyanins: cyanidin 3-rutinoside, and cyanidin 3-glucoside	[16,99,100]
	1.24 to 11.47 µmol TE/g FW	TEAC		
**Citrus fruit** (family *Rutaceae*)	2887 ± 717 µmol TE/100 g FW (orange)	ORAC	Carotenoids: violaxanthin, antheraxanthin, lutein, zeaxanthin, β-cryptoxanthin, β-carotene, α-carotene Flavonoids: naringin, neohesperidin, neoeriocitrin, kaempferol, rutin, narirutin, hesperidin, nobiletin, tangeritin, quercetin, hesperitin, naringenin, tangeretin, sinensetin, diosmin, poncirin, didymin, isorhoifolin, myricetin, resveratrol	[68,81,101,102]
	1848 ± 186 µmol TE/100 g FW (lemon),			
	1640 ± 299 µmol TE/100 g FW (grapefruit)			
	1033.8 to 1331.7 µmol TE/g (orange peel)			
	189.5 to 256.2 µmol TE/g (orange peel)	TEAC		
	0.51 to 0.68 mg/mL (orange peel)	DPPH		
	80.93 mg TE/100 g (lemon)			
	53.39 mg TE/100 g (lime)			
	69.03 mg TE/100 g (mandarin)			
	451.56 mg TE/100 g (lemon)	ABTS		
	235.38 mg TE/100 g (lime)			
	301.08 mg TE/100 g (mandarin)			
**Apple** (*Malus* *pumila*)	4592 ± 201 µmol TE/100 g FW	ORAC	Flavonoids: hyperoside, isoquercitrin, rutin, reynoutrin, avicularin, quercitrin Anthocyanins: cyanidin glycosides	[81,103,104]
	8.6 to 31.4 mmol TE/100 g (peel)			
	2.4 to 8.1 mmol TE/100 g (flesh)			
	21.9 ± 4.0 µmol of QE/100 g (no PBS wash) 17.2 ± 2.0 µmol of QE/100 g (PBS wash)	CAA		
	2.4 to 12.8 mmol TE/100 g (peel)	TEAC		
	0.8 to 2.3 mmol TE/100 g (flesh)			
	94.6 to 465.4 mg/mL (red flesh)	DPPH		
	7.4 to 22.4 TE µmol/g FW (red flesh)	ABTS		
	2.5 to 10.2 TE µmol/g FW (red flesh)	FRAP		

Note: The content in parentheses after antioxidant capacity provides additional information, including variety, tissue, or condition. In this table, TE: Trolox equivalent; DW: dry weight; FW: fresh weight; QE: quercetin equivalents; PBS: phosphate buffer saline; DM: dry matter; PCL: photochemiluminescence assay; DPPH: 1,1-dipheny1-2-picrylhydrazyl assay; TPC: total phenolic content; FRAP: ferric reducing antioxidant power assay; ORAC: oxygen radical absorbance capacity assay; TEAC: Trolox equivalent antioxidant capacity assay; PSC: peroxyl radical scavenging capacity assay; CAA: cellular antioxidant activity assay; ABTS: 2,2-azinobis (3-ethylbenzothiazoline-6-sulphonic acid) free radical scavenging assay.

## Data Availability

Not applicable.
